# Highly Dynamic and Sex-Specific Expression of microRNAs During Early ES Cell Differentiation

**DOI:** 10.1371/journal.pgen.1000620

**Published:** 2009-08-28

**Authors:** Constance Ciaudo, Nicolas Servant, Valérie Cognat, Alexis Sarazin, Emmanuelle Kieffer, Stéphane Viville, Vincent Colot, Emmanuel Barillot, Edith Heard, Olivier Voinnet

**Affiliations:** 1 CNRS UPR2357—Institut de Biologie Moléculaire des Plantes, Université de Strasbourg, Strasbourg, France; 2 CNRS UMR3215—INSERM U934, Institut Curie, Paris, France; 3 INSERM U900, Institut Curie, Paris, France; 4 Ecole des Mines de Paris, ParisTech, Fontainebleau, France; 5 CNRS UMR 8186—Département de Biologie, Ecole Normale Supérieure, Paris, France; 6 CNRS UMR7104—INSERM U964, Institut de Génétique et de Biologie Moléculaire et Cellulaire, Department of Developmental Biology, Université de Strasbourg, Faculté de Médecine, Centre Hospitalier Universitaire de Strasbourg, Illkirch, France; University of California San Francisco, United States of America

## Abstract

Embryonic stem (ES) cells are pluripotent cells derived from the inner cell mass of the mammalian blastocyst. Cellular differentiation entails loss of pluripotency and gain of lineage-specific characteristics. However, the molecular controls that govern the differentiation process remain poorly understood. We have characterized small RNA expression profiles in differentiating ES cells as a model for early mammalian development. High-throughput 454 pyro-sequencing was performed on 19–30 nt RNAs isolated from undifferentiated male and female ES cells, as well as day 2 and 5 differentiating derivatives. A discrete subset of microRNAs (miRNAs) largely dominated the small RNA repertoire, and the dynamics of their accumulation could be readily used to discriminate pluripotency from early differentiation events. Unsupervised partitioning around meloids (PAM) analysis revealed that differentiating ES cell miRNAs can be divided into three expression clusters with highly contrasted accumulation patterns. PAM analysis afforded an unprecedented level of definition in the temporal fluctuations of individual members of several miRNA genomic clusters. Notably, this unravelled highly complex post-transcriptional regulations of the key pluripotency miR-290 locus, and helped identify miR-293 as a clear outlier within this cluster. Accordingly, the miR-293 seed sequence and its predicted cellular targets differed drastically from those of the other abundant cluster members, suggesting that previous conclusions drawn from whole miR-290 over-expression need to be reconsidered. Our analysis in ES cells also uncovered a striking male-specific enrichment of the miR-302 family, which share the same seed sequence with most miR-290 family members. Accordingly, a miR-302 representative was strongly enriched in embryonic germ cells derived from primordial germ cells of male but not female mouse embryos. Identifying the chromatin remodelling and E2F-dependent transcription repressors *Ari4a* and *Arid4b* as additional targets of miR-302 and miR-290 supports and possibly expands a model integrating possible overlapping functions of the two miRNA families in mouse cell totipotency during early development. This study demonstrates that small RNA sampling throughout early ES cell differentiation enables the definition of statistically significant expression patterns for most cellular miRNAs. We have further shown that the transience of some of these miRNA patterns provides highly discriminative markers of particular ES cell states during their differentiation, an approach that might be broadly applicable to the study of early mammalian development.

## Author Summary

The discovery of the first microRNA (lin-4) in *C. elegans* in 1993 and the increasing realization that small RNAs are at the heart of many biological processes have led to a revolution in our thinking about development and disease. In animals, several hundred microRNAs (miRNAs) have been identified that regulate diverse biological processes ranging from cell metabolism to cell differentiation and growth, apoptosis, and cancer. Moreover, it has been shown that many miRNAs are characterized by highly specific spatial and temporal expression patterns supporting their role in such processes. However, the dynamics of small RNA patterns in male and female embryonic stem (ES) cells in the course of early differentiation has not been investigated so far. Our work represents the first study of this kind. Notably, we have identified new classes of miRNAs that show extremely defined temporal profiles during ES cell differentiation, as well as sex-specificity. Our results are of broad interest and importance because they raise the power of ES cells in defining the repertoire of small RNAs and their dynamics in mammals, and underline the importance of integrating miRNA expression patterns into the transcription factor networks and epigenomic maps defined in ES cells in order to provide a better understanding of the control of pluripotency and lineage commitment.

## Introduction

ES cells are pluripotent cells derived from the inner cell mass of the mammalian blastocyst. Depending on culture conditions, these cells can differentiate into various cell types [1]. Cellular differentiation entails loss of pluripotency and gain of lineage-specific characteristics. However, the molecular controls that govern the differentiation process are poorly understood. During differentiation, lineage-specific transcription factors activate the expression of specific sets of genes to form hierarchical transcription networks [2], while repressors and epigenetic modifications restrict pluripotency and help to define developmental potential [3]. Nevertheless, the precise molecular pathways involved remain unclear.

Over the past two decades, several important studies have implicated regulatory non-coding RNAs in the control of gene expression during development [4],[5]. In particular, a large body of work in several organisms has demonstrated that transcriptional regulation is controlled not only by protein factors, but also by small endogenous RNA molecules of ∼19–23 nucleotides (nt) in length called microRNAs [6]. miRNAs serve as regulators of gene expression by partially binding to complementary sites on their target transcripts. Modes of miRNA action include endonucleolytic cleavage of target mRNA, accelerated mRNA decay or repression of translation [7]–[9]. In animals, several hundred miRNAs have been identified, that regulate diverse biological processes ranging from cell metabolism to cell differentiation and growth, apoptosis, cancer and immune responses [10],[11]. Moreover, it has been shown that many miRNAs are characterized by highly specific spatial and temporal expression patterns supporting their role in such processes [12]. The biogenesis of miRNAs involves nuclear processing of a long primary transcript (pri-miRNA) into a stem-loop structured pre-miRNA by the RNase III Drosha. The pre-miRNA is then exported to the cytoplasm and further matured by the RNase III Dicer into a siRNA-like duplex. The single-stranded mature miRNA is then asymmetrically transferred into an Argonaute-containing miRNA effector complex, while the passenger strand, or microRNA* (miR*) is degraded [13]–[16].

Several recent reports underscore important roles for miRNAs in preventing differentiation of ES cells, most notably through the activity of the pluripotency miR-290 cluster [17]–[20]. However, the dynamics of small RNA accumulation during early ES cell differentiation, particularly at early stages, has not been investigated so far. Additionally it remains unclear whether expression of some small RNAs can be regulated in a sex-specific manner and could thereby contribute to poorly understood processes such as X chromosome inactivation in females (for a review, see [21]). To address these issues, we have analysed mouse ES cell small RNA populations through the generation and sequencing of small RNA libraries isolated from both male and females cell lines at day 0, 2 and 5 of differentiation. We describe here the results of this analysis, focusing on the most abundant class of isolated small RNAs, the miRNAs.

## Results/Discussion

### Cloning of small RNA from differentiating male and female ES cells

In order to examine the small RNA profiles of mouse ES cells during early differentiation, we generated six libraries using RNA isolated from male (E14, XY1) and female (PGK, XX1) ES cells, either in the undifferentiated state (D0), or after 2 or 5 days of differentiation (D2, D5, respectively). These ES cell lines are cultured under feeder-free conditions in the undifferentiated state, thus avoiding any contamination of fibroblast cell-derived small RNAs. We induced differentiation by LIF withdrawal and cell dilution (see Materials and Methods), which differs from most previous studies [17],[22],[23], where differentiation had been induced by retinoic acid (RA) that preferentially promotes neuronal differentiation. By initiating a slower differentiation process than with RA treatment, we hoped to be able to monitor the diversity of early events during the acquisition of cell identity. We confirmed the early differentiation status of the cells throughout time by monitorig the expression of key differentiation and cell fate-specific markers (Figure S1).

Libraries were prepared using the ∼19-to-30 nucleotides (nt) fraction of total RNA from ES cells and differentiating cells (see Materials and Methods). This fraction was chosen, as it represents the size range of known small RNAs families in mammals [24]. To obtain a comprehensive picture of the corresponding small RNA profiles, 50,000-100,000 reads per library were produced using the 454 pyro-sequencing technology. For all six libraries, initial analysis of the cloned populations revealed that the vast majority of small RNAs present in these samples were 22–23 nt in length (Figure S2A). Bioinformatics analyses employing available small RNA databases (see Materials and Methods) showed that most small RNAs corresponded to known miRNAs, while the remaining, less abundant classes corresponded to ribosomal RNA (rRNAs), transfer RNA (tRNAs), and other non-coding (nc)RNAs. An additional small RNA class mapped to repeated (repeat) and non-repeated (genome) genomic DNA, while a final fraction corresponded to unclassified species, with no obvious matches to the mouse genome (not annotated) (Figure S2B). The overall distribution of small RNAs did not vary significantly between male and female ES cell differentiation. However, the cloned miRNA fraction significantly increased during differentiation (45% and 65% of cloned sequences at D0 and D5, respectively; Figure S2B), suggesting a highly dynamic regulation of this population of small RNAs during early differentiation. Given the critical role of miRNAs in regulating differentiation events during development [25], we decided to focus on this specific class of small RNAs.

Libraries were queried against precursor sequences of all known microRNAs, as deposited in miRbase (release 10.1, December 2007). The complete set of cloned miRNAs is presented in Dataset S1 (Sheet 1), alongside the cloning frequency relative to the total number of miRNAs in each library. The results were filtered according to a two-component Gaussian mixture model (Figure S3 and Materials and Methods) defining a lower threshold frequency of 0.05%, below which cloned miRNAs were considered as background and removed from the dataset, apart from a group of 17 novel miRNAs* not previously cloned or deposited on miRbase release 12.0 (designated by ‘SL’ in Dataset S1, Sheet 2). The identification of these miRNA*, which are transient intermediates in the miRNA biogenesis pathway, demonstrated that our cloning/sequencing approach was of adequate depth.

In order to assign quantitative miRNA measurements to different mouse chromosomes we used an adaptation of the VAMP software (Visualisation and Analysis of Molecular Profiles; Figure S4) [26]. For the analysis of miRNA expression patterns during differentiation, we were able to treat male and female cell samples as expression replicates, as the Pearson and Spearman correlation coefficients between miRNA quantification and sequencing frequencies indicated that male and female cell libraries are highly comparable at each differentiation time point (p-values<2.2^e-16^; Figure S5 and Materials and Methods). A lower (albeit significant) Pearson score at D5 is likely due to the fact that sex-enrich miRNA are fully expressed at this stage and create differences between male and female samples (see later in the text). The lower Spearman test score obtained at D0 is likely explained by the fact that few miRNA clusters are expressed in totipotent cells (Dataset S1, Sheet 1; see below).

### Time-course analysis of abundant ES cell miRNAs discriminates pluripotency from early differentiation

**Figure 1:**
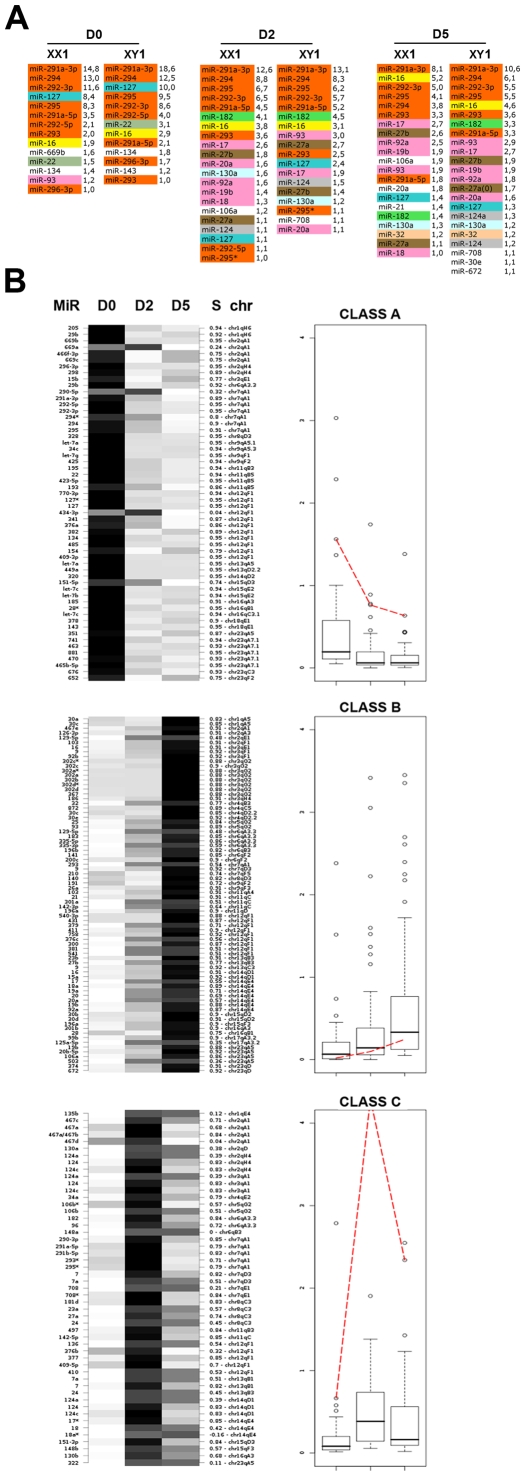
A discrete set of miRNAs dominates the small RNA repertoire. (A) microRNAs with cloning frequencies >1% are represented in male (E14, XY1) and female (PGK, XX1) ES cells at the indicated time points. microRNAs belonging to the same family are highlighted using the same color code. Individual expression percentages are indicated for each microRNA. (B) Partitioning clustering analysis (PAM – partitioning around medoids) of miRNA expression data. The miRNAs were grouped into 3 clusters (A, B, and C) according to their expression profile. The high silhouette (S) score observed for most miRNA—ranging normally from −1 (bad) to +1 (good)—demonstrates the validity of the PAM classification approach. The red dotted line represents the medoid of each class (see Materials and Methods for further details). The data are from the female ES cell line PGK (XX1) and the male ES cell line E14 (XY1), which were treated as biological replicates according to the results of Pearson and Spearman correlation analyses.

In our samples only a discrete set of miRNAs dominates the small RNA repertoire of undifferentiated and early differentiating ES cells (miRNAs with cloning frequencies >1%; Figure 1A), which agrees with the results of previous studies [17],[22],[27]. However, unlike in previous work attributing an ES-cell specific status to miR-21 [22], this miRNA was cloned at a low frequency in all our samples (1,4% in female at D5, <1% in all other samples). In fact its levels even appear to increase slightly during early differentiation at D2 (Figure 1A; Dataset S1).

Additionally, miR-15b and miR-16, which were among the most abundant small RNAs identified by Calabrese *et al.* in undifferentiated mouse ES cells, were either not (miR-15b) or moderately (miR-16) represented at D0 in our experiments. In fact, the levels of miR-16 increased steadily throughout differentiation and it was one of the most abundant miRNA in male and female cells at D5 (Figure 1A), clearly indicating that miR-16 is not a reliable indicator of ES cell pluripotency. Furthermore, elevated (>1%) expression of the oncogenic miR-17_92 cluster was only apparent in our D2 (7%) and D5 (10.5%) samples (Figure 1A), but not in undifferentiated ES cells, unlike in the previous study where members of this cluster were cloned at a frequency of 11% from undifferentiated cells [22].

Most of these discrepancies can be readily explained by the fact that the ES cell lines and differentiation conditions used in these previous studies were different to ours. The fact that we did not use RA as a differentiating agent, which is known to induce expression of specific sets of microRNAs targeting Nanog, Oct4 (also known as Pou5f1) and Sox2 coding sequences [28], could account, for instance, for the low accumulation of specific miRNAs in our samples, including miR-15b. Our differentiation protocol also induced slow differentiation of specific lineage found in mouse embryo as presented in Figure S1 with little variation between the two ES male and female ES cell lines tested. Secondly, our ES cells were grown using feeder-free conditions, while other studies employed embryonic fibroblasts feeder cells for the culture of ES cells, and we note that miR-21 is one of the most abundant miRNAs found in fibroblasts [27]. Furthermore, a distinct analysis of SOLiD-based small RNA sequencing data obtained from an independent XY ES cell line grown on feeders indeed showed a significant enrichment (5%) of cloned miR-21 at D0 (C.C, N.S, E.B and H.E; data not shown). Therefore, the presence of miR-21 is unlikely to be a genuine feature of undifferentiated mouse ES cells.

In some of the previous studies, the use of feeder cells also imposed a lag phase of about 24 h following their removal by adsorption. Such a procedure may induce the very earliest steps of ES cell differentiation. Indeed, we note that the miRNA profile of our D2 samples is remarkably similar to that reported by Calabrese and colleagues, raising the possibility that their analysis involved a mixture of very early differentiating and undifferentiated ES cells, rather than *bona fide* undifferentiated cells. This would also explain why miR-22 -which we cloned at a high frequency specifically in both male and female undifferentiated samples- was not overrepresented in their study.

Our high resolution time-course analysis of the most abundant miRNAs from two distinct mouse ES cell lines has enabled to discriminate pluripotency patterns from early differentiation patterns. Notably, the miR-290_295 cluster, miR-127 and miR-22 contribute collectively to more than 65% of all cellular miRNAs of undifferentiated ES cells, and their respective abundance consistently decreases during early differentiation. Similar figures were also obtained in two additional, independent analyses employing the SOLEXA and SOLiD deep sequencing technologies (data not shown). We propose, therefore, that these molecules represent reliable small RNA markers of pluripotency. We further distinguish abundant miRNAs present in undifferentiated cells, the expression of which increases during differentiation (exemplified by miR-16), from those that are initially only moderately or poorly expressed at D0, but are highly abundant by D2 and D5 of differentiation (Figure 1A). The latter includes the well-characterized miR-17_92 cluster, which targets several tumor suppressors and is enriched in many types of cancer (for a review, see [29]), but also members of the miR-27 family, which suppresses expression of the breast cancer marker CYP1B1 [30]. Such co-expression of pro- as well as anti-oncogenic miRNAs might ensure that processes favoring cell proliferation versus acquisition of cell identity are appropriately balanced in early differentiating ES cells.

### miRNA classes revealed by partitioning around medoids (PAM) analysis include high but also many low-to-moderate abundance miRNAs, of which many correlate with genomic clustering

There is currently no predictable correlation between the level of miRNA accumulation and their efficiency in suppressing gene expression. On the one hand, only a fraction of miRNAs that accumulate at saturating levels (eg the miR-290 cluster) might effectively recruit miRNPs for target regulation. On the other hand, moderately expressed miRNAs might be present at sufficient levels to suppress low-abundance transcripts. To address this issue objectively, we used a clustering analysis based on partitioning (Partitioning Around Medoids, PAM (Kaufman & Rousseeuw, 1990; see Materials and Methods and Figure 1B) that groups all miRNAs cloned at a statistically significant frequency (>0.05%) into classes with correlated expression profiles. miRNAs in differentiating ES cells can be divided into three highly contrasted expression clusters designated A, B and C (Figure 1B), which can be further refined into 10 sub-groups, detailed in Figure S6 (See also Materials and Methods for details). miRNAs in class A are present in undifferentiated ES cells (D0), but progressively decrease in abundance as differentiation proceeds. miRNAs grouped into class B show an inverse pattern to cluster A, i.e. an increase in abundance during differentiation. Class C, on the other hand, is characterized by a peak of expression at Day 2, followed by a decrease at Day 5. We believe that these expression patterns are of biological relevance, because many miRNAs known to be neighbours along the genome (and thus likely to be co-regulated) were grouped together within the same PAM classes (Figure 1B, Figure S6). Further strengthening this idea, use of an *in silico* boostraping procedure employing a randomized miRNA expression dataset indicated that the observed PAM clustering in the real data set cannot occur by chance (Figure S7, Materials and Methods). Moreover, we experimentally verified that representative members of the 3 major PAM classes (chosen not to be in the same miRNA genomic clusters) do indeed exhibit the predicted expression profile (Figure S8).

Therefore, analysis of discrete expression subclasses (as presented in Figure S6) should provide an important handle with which to link distinct miRNA gene families functionally, since their coordinated expression likely entails the targeting of related functions within common cellular pathways. This analysis also reveals for the first time that miRNA temporal expression patterns can be extremely narrow, as illustrated with members of PAM class C, epitomized here by miR-182 and miR-27a (Figure 1A–1B and Figure S8). The unexpected peak of expression at Day 2 for this class of miRNAs could not have been appreciated in previous, single time-point analyses. This type of transient expression pattern must be linked to early loss of pluripotency and/or initiation of lineage-specific expression pathways and such miRNAs merit future investigation for their functions in early mammalian development. Additionally, they represent new and useful markers for early ES cell early differentiation.

### Extreme temporal variations in the accumulation of individual members of two highly expressed miRNA clusters

Our time course analysis during differentiation provided us with a unique opportunity to dissect the specific regulation of individual members of genomically clustered miRNA genes. We first focused our attention on the highly expressed miR-290 cluster located on chromosome 7, a potent marker of mouse ES cell pluripotency (Figure 1A–1B) [17],[22]. The mouse miR-290 cluster was recently shown to target several key cell cycle regulators and transcriptional repressors to enable rapid G1-S transition and maintenance of DNA de-methylation, two defining features of stem cells [19],[31]. Interestingly, both studies involved the rescue of either Dicer^−/−^ or Dgcr8^−/−^ ES cell defects through ectopic expression of the entire miR-290 cluster or some of its highly abundant members [20],[32]. These and other studies thus point to the effects of the miR-290 cluster being due to a single, coordinated expression unit with functionally redundant products.

**Figure 2:**
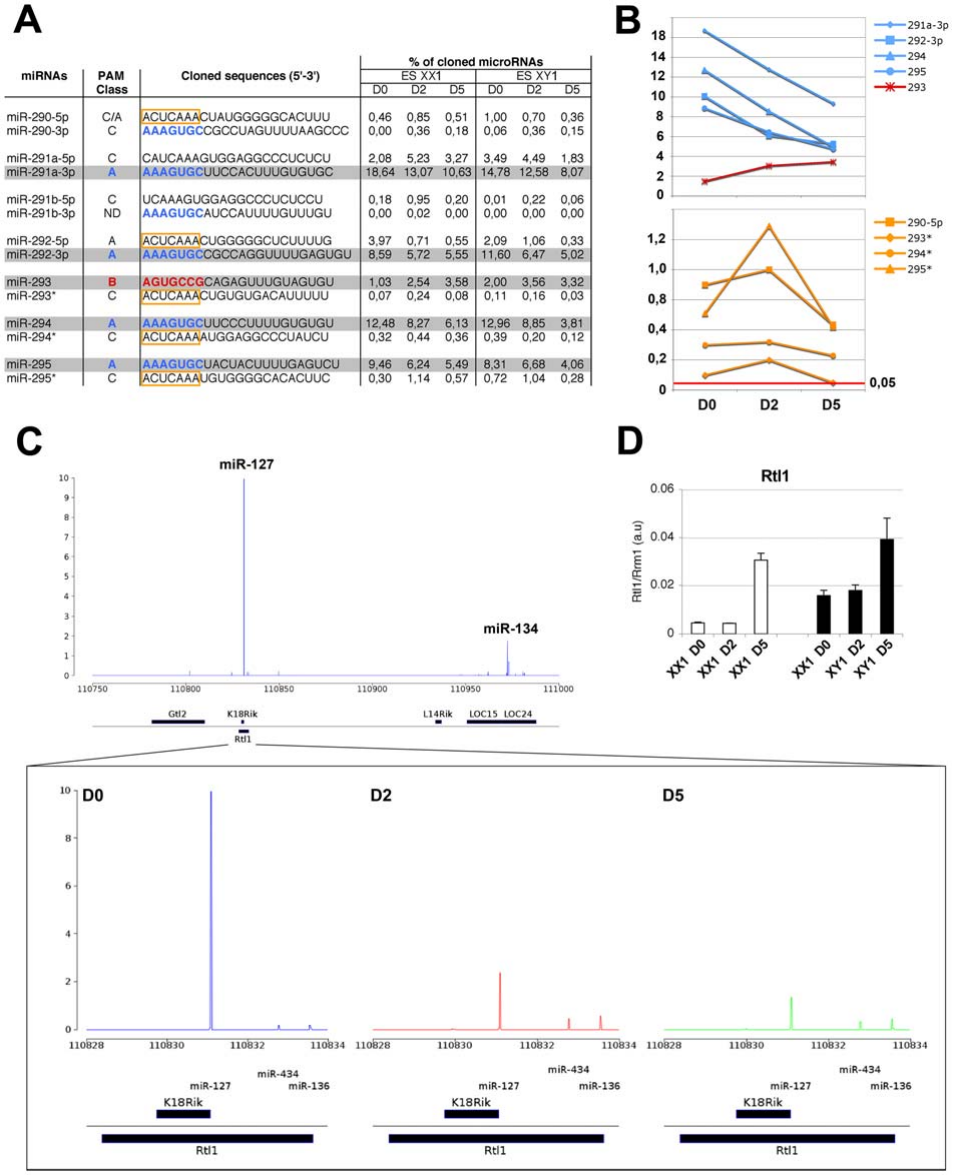
Extreme temporal variations in the accumulation of individual members of two highly expressed miRNA clusters. (A) The table shows the individual cloning frequencies (expressed as a percentage of total cloned miRNAs) of the various miR-290_295 family members, as assessed throughout differentiation of male (E14, XY1) and female (PGK, XX1) ES cells. The seed sequences are highlighted in color (blue or red), and PAM classes of individual microRNAs are provided. The most abundant miRNAs are shaded in grey. (B) Graphic representation of the highly expressed members of the miR-290 family (as depicted in (A)) and their corresponding miRNA* sequences showing contrasted expression profiles. The data are averaged from the XY1 and XX1 values presented in (A). (C) Visualization of the expression of miRNAs found within the *Rtl1* locus on chromosome 12, in undifferentiated ES cells. The inlay shows the expression dynamics of the 3 miRNAs present on the opposite strand of the *Rtl1* mRNA throughout ES cell differentiation. This unravels a major contribution of miR-127. (D) Quantitative-RT-PCR analysis of *Rtl1* expression in male and female ES cell samples. The data are from the female ES cell line PGK (XX1) and the male ES cell line E14 (XY1). They were treated as biological replicates in (B) and (C), according to the results of Pearson and Spearman correlation analyses.

In agreement with these previous studies, separate time-course analyses of each member revealed that only 4 miRNAs, which share the same AAAGUGC 5′ seed sequence (miR-291a-3p, miR-292-3p, miR-294, miR-295, Figure 2A, blue), likely contribute significantly to the global trend of miR-290 cluster expression (reduced throughout differentiation, PAM class A; Figure 2A, grey). Nonetheless, PAM classification of individual miRNAs revealed sharp differences in temporal expression between some members of the cluster, and, more unexpectedly, between the mature and presumed passenger strand sequences of the most abundant miRNAs, of which three out of four were grouped into PAM class C rather than PAM class A (Figure 2B). Moreover, these 3 presumptive miRNA* and the class C miR-290-5p all have the same 5′ seed sequence ACUCAAA(C/A), a feature also shared by the class A miR-292-5p (Figure 2A, yellow boxes). This common characteristic, together with their cloning frequencies being well above background (e.g. miR-295*), suggests that several of these small RNA might be specifically engaged into a common regulatory pathway at D2 of differentiation. In agreement with this hypothesis, widespread functional recruitment of miRNA* has been inferred in *Drosophilid*, and several specific cases were recently experimentally validated *in vitro* and *in vivo* [33].

Perhaps even more compelling, the analysis of the miR-290 cluster also revealed an unexpected expression profile for the highly abundant miR-293. This miRNA shows the opposite pattern to all of the other highly expressed members of the miR-290 cluster (increase throughout differentiation, PAM class B, Figure 1B), suggesting drastically distinct targets and cellular functions for this specific miRNA. Indeed, a gene ontology (GO) analysis (http://www.mirz.unibas.ch/ElMMo2/) of predicted target transcripts revealed a consensus set of regulated cellular functions for miR-291a-3p, miR-292-3p, miR-294 and miR-295, but not for miR-293 (Dataset S2 for the EIMMo target prediction software and Dataset S3 for the Pictar target prediction software). Accordingly, a seed inspection uncovered a completely different sequence for miR-293 (Figure 2A, red), thereby confirming its singular status within the miRNA-290 cluster. A recent study also showed that several miRNA of the miR-290 cluster could individually help reprogramming mouse fibroblasts into induced pluripotent cells [34]. However, this could not be achieved with miR-293, indicating different functions for this specific miRNA. The miR-290 cluster comprises two pri-miRNA giving rise to six pre-miRNAs, of which pre-miR-293, premir-294 and premiR-295 are produced from the same primary transcript [17],[18]. Thus, the most likely explanation to the result obtained in our analysis is a specific, post-transcriptional regulation of pre-miR-293 or mature miR-293. In any case, these results show that the contribution of the miR-290 cluster to pluripotency cannot be interpreted in terms of a single, coordinated expression unit with redundant products.

Collectively, these data reinforce the growing view that miRNA genes undergo extensive post-transcriptional regulation through mechanisms that selectively affect pri-miRNA processing and/or pre-miRNA stabilization [35], notwithstanding possible effects on mature miRNAs, as recently suggested in plants [36]. These refinements in gene expression suggest that the regulatory potential and versatility of miRNAs is likely much broader than initially anticipated. Given its abundance, single chromosomal location and well-defined composition, studies of the miR-290 cluster in the ES cell-based system described here could help addressing these important issues further.

The second most highly expressed miRNA cluster in undifferentiated ES cells is located on chromosome 12 and contains a total of 26 members, as annotated in miRbase (Figure 2C). Among these, a short cluster of maternally expressed miRNAs genes (miR-431, miR-433, miR-127, miR-434 and miR-136) is transcribed and processed from an antisense gene to the paternally expressed Retrotransposon-like 1 (*Rtl1*) gene, the recently characterized protein product of which appears to be indispensable for mouse foetal development [37]. Due to their perfect complementarity to *Rtl1*, the above miRNAs have been proposed to mediate *trans*-allelic RNAi at the *Rtl1* locus in a variety of mouse embryonic tissues, based on Northern and 5′ RACE analyses {Seitz, 2003 #930; Davis, 2005 #918}. However, their respective contribution to *Rtl1* silencing has not been addressed. Our time-course analysis revealed that of these five miRNAs, only miR-127, miR-434 and miR-136, are likely to contribute to *Rtl1* silencing in ES cells (Figure 2D) because their cloning frequencies largely exceeds that of the other members of the cluster, which accumulate at background level (Figure 2C, Dataset S1). The three miRNAs can be further distinguished based on their expression profiles and respective cloning frequencies, with miR-127 contributing alone 10% of all cloned miRNAs at D0. Unlike miR-434 and miR-136 (PAM class C), miR-127 expression gradually decreases during the differentiation process (PAM class A), a pattern inversely correlated to that of *Rtl1* expression in ES cells, as assayed by Quantitative Reverse-transcriptase PCR (Q-RT-PCR; Figure 2D). We conclude that miR-127 is likely the major contributor of *Rtl1* silencing in differentiating mouse ES cells. Thus, in contrast to the complex situation described above for the miR-290 cluster, in this particular case the PAM analysis of temporal miRNA/target variations shows that the effect of a *miRNA* gene cluster can be probably equated to that of a single miRNA member within it.

### Male-specific regulation of the miR-302 gene in embryonic stem cells and germ cells

**Figure 3:**
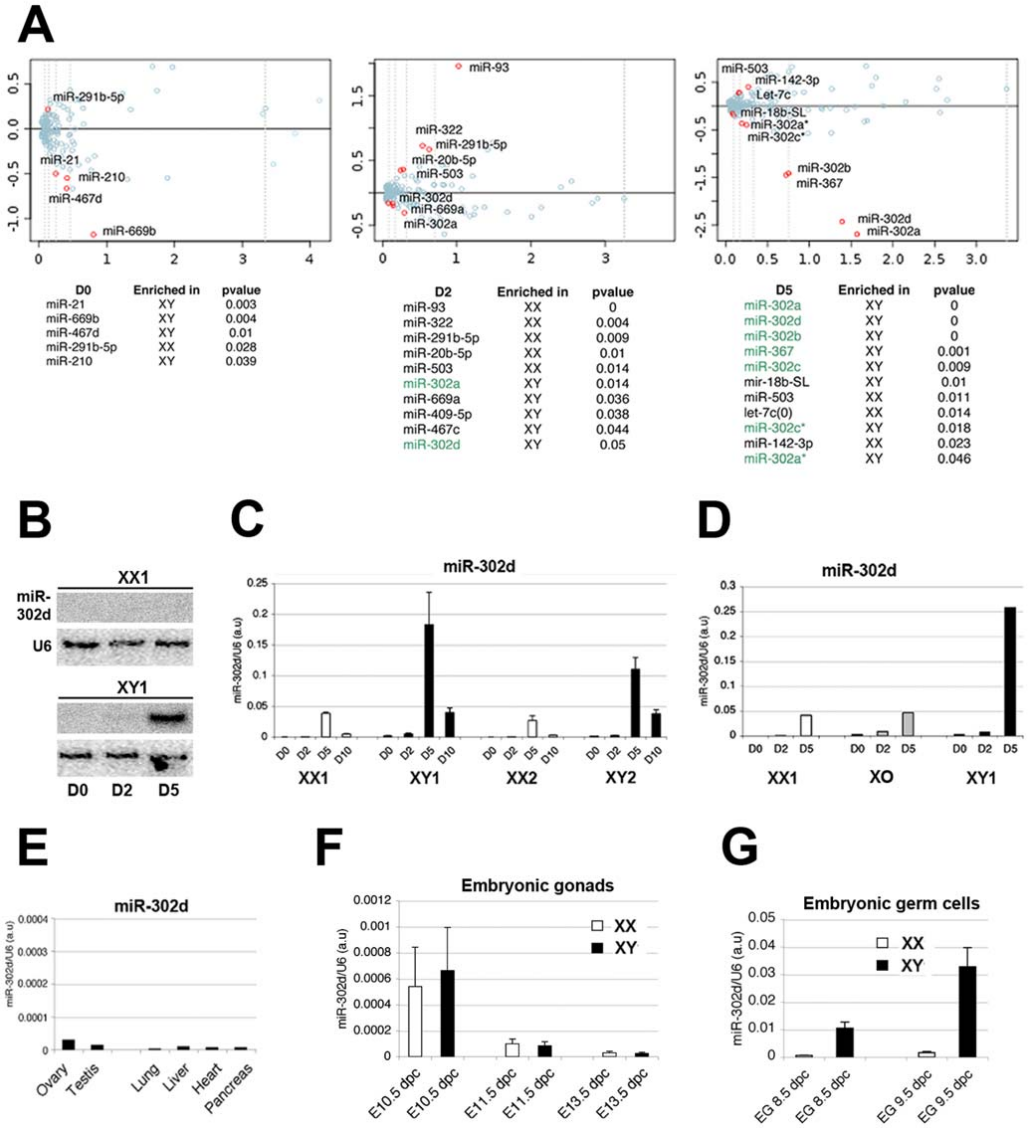
Male-specific regulation of the miR-302 gene in embryonic stem cells and germ cells. (A) MA Plot of microRNAs singularizes those enriched in male (E14, XY1) or female (PGK, XX1) cells at different time points, as indicated in red (see Materials and Methods for details). (B) Northern Blot analysis of miR-302d in female (XX1; PGK) and male (XY1; E14) cell lines at D0, D2, and D5 of differentiation. (C,D) Quantification by Q-RT-PCR analysis of mature miR-302d in two independent female (PGK, XX1 and LF2, XX2) and male (E14, XY1 and HM1, XY2) ES cell lines at D0, D2, D5, and D10 of differentiation (C) and in the X^PGK^O ES cell line (D). (E) Quantification by RT-PCR of miR-302d accumulation in various adult mouse tissues. (F,G) Quantification by RT-PCR of mir-302d expression in male (XY, black) and female (XX, white) gonads at 10.5 to 13.5 dpc (F) and 8.5 to 9.5 dpc. (G) Same as in (F), but in male (XY, black) and female (XX, white) embryonic germ cells (EG).

One goal of this study was to identify putative sex-specific small RNAs, including miRNAs. To this end, we reanalyzed separately the data in male and female ES cell samples, in order to set apart outlier miRNAs the distribution of which diverged significantly from the median obtained upon analysis of both sexes (Figure S5). Few miRNAs were found to be differentially expressed between sexes (using MA plot transformation see Materials and Methods); and for those that were, their level tended to increase with differentiation (Figure 3A). Although some of these variations in expression between male and female ES cells could be attributed to differences in the rates of differentiation between the ES cell lines used, the greatest and most striking difference between the two sexes was observed at D5 with the miR-302 genomic cluster. For this cluster, all five members (including some miRNA* sequences; Figure 3A, highlighted in green) were cloned at a significant and much higher frequency in male (>10% of all miRNAs cloned) as opposed to female D5 differentiated ES cells (<0.5% of all miRNAs cloned, Figure 3B; Dataset S1). This suggests a strong and specific transcriptional enhancement of the miR-302 gene in male cells. Northern analyses of total RNA confirmed that the levels of miR-302d (one of the most frequently cloned representatives of the miR-302 cluster, Figure 3B, upper panel) are at least 20 fold higher in D5 male cells than in D5 female cells (Figure 3B, lower panel). Further support for a male-specific enrichment of the miR-302 family came from Q-RT-PCR analyses showing that X^PGK^O ES cells (lacking a Y or a X chromosome) displayed only a very minor increase in miR-302d content, similar to female XX cells at D5 (Figure 3D). We could also rule out possible cell line-specific effects, because Q-RT-PCR analyses of mature miRNAs from two independent male (E14; XY1 and HM1; XY2) as well as two independent female (PGK; XX1 and LF2; XX2) cell lines gave similar results (Figure 3C). This Q-RT-PCR analysis also revealed that in differentiating female samples, miR-302d could also be detected, albeit at much lower expression levels. To examine further this male-specific differentiation miR-302 expression pattern, RNA from ES cells that had undergone differentiation for 10 days was also examined. This revealed that the increase in miR-302 observed at D5 in males is transient because it had decreased by D10 (Figure 3C). This highly dynamic and apparently sex-specific pattern suggested that the miR-302 family might have a role during a narrow window of male development.

To investigate this possibility in vivo, we monitored mirR-302d expression in various tissues of adult mice (Figure 3E). However, expression of this cluster was at or below detection limit of Q-RT PCR analysis in all sampled tissues (Figure 3E). Based on the strong enrichment of miR-302 at Day 5 of early differentiation and its male ES cell-specificity, we thus compared its accumulation in dissected gonads of male and female embryos from 13.5 to 10.5 dpc (Figure 3F). No sex-related difference could be detected at these time points, although miR-302d expression was clearly higher at 10.5 dpc in both males and females, and reached background levels at 13.5 dpc (Figure 3F). These results thus suggested that any putative sex-specific embryonic expression of miR-302 should occur at earlier stages, before colonization of the gonads by primordial germ cells (PGC), which are also known to form during early ES cell differentiation (for a review, see [38]). To address this issue, we analyzed miR-302 expression in male and female pluripotent embryonic germ cells (EG) derived from PGCs that had been isolated at various stages of embryogenesis (8.5, and 9.5 dpc). Indeed, while it was at background levels in female samples, a strong male-specific enrichment of miR-302d was observed in EG cell lines at both stages, and particularly at 9.5 dpc (Figure 3G). Our ES cell differentiation analysis thus uncovers the first example of sex-specific regulation of a mammalian miRNA.

### The chromatin remodelling and E2F-dependent transcription repressors Arid4a and Arid4b are putative targets of miR-302

A recent study indicates that members of the miR-290 and of the oncogenic miR-17_92 cluster are among the most abundant miRNAs found in proliferating mouse PGCs [39]. Although this study involved a mixture of male and female embryos and thus, could not identify the sex-specific enrichment of miR-302 members, this profile resembles that of D2 and D5 female ES cells in our early differentiation system. Interestingly, all members of the miR-302 cluster share the same AAGUGC(U/C) 5′ seed sequence with the highly expressed members of the miR-290 cluster (with the notable exception, of course, of miR-293; Figure 2A–2B). It has thus been speculated that the two clusters carry out similar functions, particularly in totipotency, as shown in human ES cells [40]. This idea was recently given some experimental support by the demonstration that certain proliferation defects of Dgcr8^−/−^ mouse ES cells are rescued to a similar extent through ectopic expression of individual members of either the mouse miR-302 or miR-290 clusters [32]. These observations together with the results of the present study thus predicted that some shared targets of the miR-302 and miR-290 clusters should be specifically downregulated in male embryonic stem and germ cells, in which the miR-302 family accumulates at much higher levels than in female cells.

**Figure 4:**
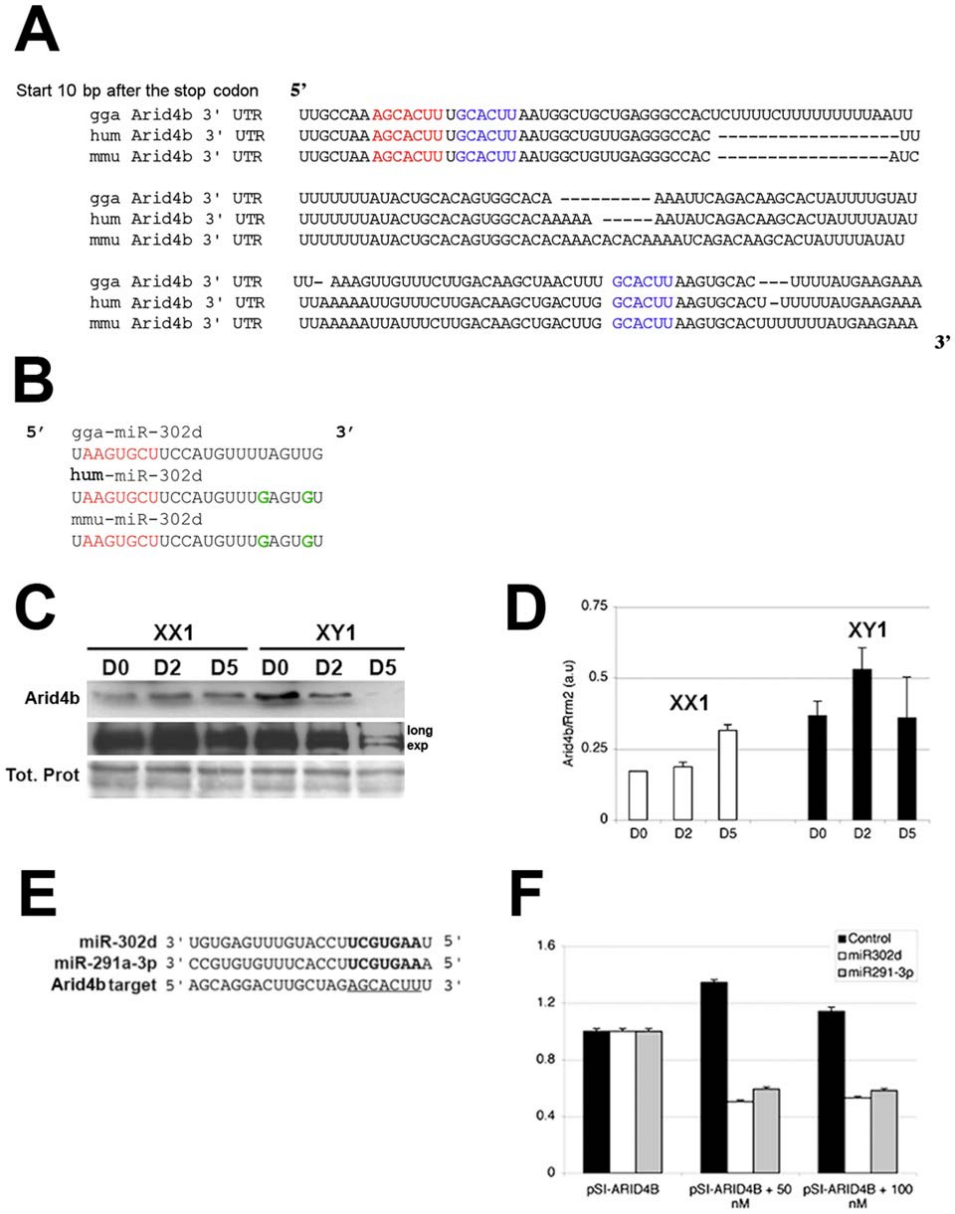
Male-specific down-regulation of the chromatin remodelling and E2F-dependent transcription repressor Arid4b in mouse ES cells. (A,B) High conservation of the 3′UTR of the Arid4b gene (A) and of the miR-302d seed (B) between chicken (gga), mouse (mmu), and human (hum). The predicted target sites of miR-302 are indicated in red (7-mer) and blue (6-mers). The green lettering in (B) depicts sequence polymorphism between chicken and mammals. (C,D) Expression levels of Arid4b protein (C) and mRNA (D) in male (XY1, E14) and female (XX2, LF2) ES cells during differentiation. (E) Arid4b is a putative target of both miR-302 and miR-290 families, as shown by identical seed matches. (F) Results form a standard dual luciferase-based miRNA repression assay employing the Arid4b 3′UTR fused to luciferase (psiCHECK-Arid4b). HEK-293 cells were transiently transfected with psiCHECK-Arid4b together with miR-302d, miR-291-3p, or a control siRNA, using lipofectamine 2000 (Invitrogen). Luciferase activity was measured 24 h post transfection.

To identify cellular targets of the miR-302 family, we used two different algorithms: Pic-Tar [41] and the EIMMo microRNA target prediction server (http://www.mirz.unibas.ch/ElMMo2/). These softwares search mRNA 3′-UTRs for the presence of conserved 7-mers matching the seed region of queried miRNAs. Strikingly, both algorithms identified the 3′-UTR of the *Arid4a* and *Arid4b* paralogous genes as first ranking candidates (Dataset S2 and Dataset S3). Other high-scoring candidates included a set of genes that had been previously validated as targets for miR-290 in mice. Both human *ARID4* paralogs were previously known as retinoblastoma-binding protein 1 (RBBP1 or RBP1) [42]. They serve as adapters to recruit the mSin3A-Histone deacetylase (HDAC) to E2F-dependent promoters undergoing transcriptional repression by Rb [43]. We decided to focus on the *Arid4b* gene, whose 3′-UTR contains three evolutionary conserved matches for the seed shared by miR-302 and miR-290 (Figure 4A–4B). Western Blot analysis at D0, D2 and D5 revealed a steady accumulation profile for the Arid4b protein throughout early differentiation of female mouse ES cells (Figure 4C, left panel), in which miR-302 levels remained low (Figure 3B–3C). In contrast, there was a progressive loss of Arid4b accumulation in male cells (Figure 4C, left panel), a pattern inversely correlated to that of miR-302d levels (Figure 3B–3C). Q-RT-PCR analyses showed that slight changes in *Arid4b* mRNA levels were observed throughout differentiation of male cells. However, these slight changes could not account for the strong reduction in Arid4b protein levels in the same samples, thereby indicating an effect at the protein level consistent with a seed/3′-UTR type of regulation by the miR-302 family.

To validate the predicted interaction between miR-302 and *Arid4b*, we used a dual luciferase-based reporter assay. The entire 3′-UTR of the endogenous *Arid4b* mRNA was inserted dowsntream of the open reading frame of a Renilla luciferase reporter gene. Expression of the resulting construct was then measured in transfected human HEK-293 cells, which are devoid of miR-302 (data not shown). The authentic miR/miR* duplex of miR-302d was then chemically synthesized and transfected into cells together with the reporter plasmid. An unrelated siRNA duplex was transfected in parallel, as a negative control. We also used a miR-291a-3p duplex as a representative of the miR-290 family, of which several members are also predicted to target *Arid4b* owing to seed identity with miR-302 (Figure 4D, Dataset S2 and Dataset S3). 24 h post-transfection, a similar decrease in Renilla luciferase reporter gene activity was observed with both miR-302d and miR-291a-3p treatments, but not upon transfection of the control siRNA duplex (Figure 4D), indicating a sequence-specific effect. Together with our ES cell time-course analysis (Figure 3B and 3D; Figure 4C) these results strongly suggest (i) that *Arid4b* is a common mRNA target of miR-302 and miR-290 family members and (ii) that the differences in Arid4b levels between male and female D2 and D5 ES cells are due to the male-specific accumulation of miR-302. By extension, it can be inferred that many common targets of miR-302 and miR-290 are likely to undergo similar differences in expression in tissues or cell types showing sex-related polymorphism of miR-302 expression, including EGs (Figure 3G) and, presumably, PGCs. One possible explanation to this male-specific pattern is that it might be generated by the subpopulation of male ES cells that goes down the germline differentiation pathway. Thus, it could reflect an important male-specific genetic program, possibly normally initiated in male PGCs. However, testing this idea in ES cells will first require the isolation of germline cells within the population, which, though technically challenging, represent an interesting perspective of the present work.

Members of the miR-302 and miR-290 clusters can individually rescue the proliferation defects of mouse *Dgcr8^−/−^* ES cells [32]. In this context, identification of *Arid4* as a novel target of both miR-290 and miR-302 is entirely consistent with the established role of ARID4 as an Rb-mediated repressor of E2F-dependent transcription, which is mandatory for the G1-S phase transition in the cell cycle [44]. These data can now be assembled into a comprehensive, albeit still speculative model, integrating the possible overlapping functions of miR-290 and miR-302 in mouse cell totipotency during early development (Figure S9).

This study demonstrates that small RNA sampling throughout early ES cell differentiation enables the definition of expression patterns for most cellular miRNAs. We have further shown that the transience of some of these miRNA patterns provides highly discriminative markers of particular ES cell states during their differentiation, an approach that might be broadly applicable to the study of early mammalian development. Our study also underscores the benefit of unsupervised classification analyses in deciphering complex regulations of miRNAs cistrons, notably by uncovering outlier members within miRNA clusters, as shown here with the surprising findings made with miR-293. The analysis finally unravelled a puzzling enrichment of miR-302 expression during male ES cell differentiation as well as in male embryonic germ cells, suggesting a contribution of this miR family to male germline determination. We are currently in the process of generating miR-302 conditional-deletion ES cells in order to produce miR-302-deficient mice. Analysis of these animals, and notably of their germlines, might provide important insights into sex-specific miRNA regulations.

## Materials and Methods

### Culture and in vitro differentiation of ES cells

Female PGK (XX1) and LF2 (XX2) ES cell lines, male E14 (XY1) and HM1 (XY2) cell lines, and the X^PGK^O cell line (from Dr Neil Brockdorff laboratory) were cultured in Dulbecco's Modified Eagle Media (DMEM) (Invitrogen), containing 15% FCS (Bio West), 1000 U/ml LIF (Chemicon), 0.1 mM 2-mercaptoethanol (Invitrogen), 0.05 mg/ml of streptomycin (Invitrogen) and 50 U/ml of penicillin (Invitrogen) on a gelatin-coated support in the absence of feeder cells. The 4 EG cell lines (have been derived in Dr Stephane Viville laboratory) were cultivated with feeder cells under the same conditions. Differentiation was induced spontaneously into LIF-free DMEM, 10% FCS medium, at day 2, 5 and 10 of differentiation. The culture medium was changed daily. All cells were grown at 37°C in 8% CO2.

### RNA library preparation and analysis

Total cellular RNA from XX1 and XY1 cell lines at D0, 2, 5 was prepared using Trizol reagent (MRC Molecular Research Center) following the manufacturer's instructions. Small RNA cloning was performed as described in [45] using 200 µg of total RNA per library. Libraries were sequenced using the 454 technology (http://www.454.com). Sequences were annotated with blast (word size = 7/no filter) using the following databases as references. Genomic sequences were retrieved from release mm9 of the mouse genome from the UCSC Genome Browser database (NCBI build 37, July 2007). tRNA, rRNA, and other non-coding RNA sequences were extracted from release 158 of Genbank (February 15, 2007), microRNA precursor sequences were extracted from miRbase (release 10.1, December 2007). The results were filtered to authorize 0, 1 or 2 mismatches per small RNA sequence, to take into account polylorphism and sequencing errors.

Northern blot analysis was as described [46]. 30 µg of total RNA were used per lane. Hybridization probes corresponded to 5′ ^32^P-radiolabelled oligodeoxynucleotide complementary to the miR-302d sequence or to part of the U6 snRNA sequence (used as loading control). Blots were analyzed and quantified by phosphorimaging (FLA7000 scanner; Fuji).

### Real-time PCR gene expression analysis

Real-time PCR reagents for miRNAs and control U6 snRNA were from Qiagen. For RT reactions, 1 µg total RNA was reverse transcribed using the miScript Reverse Transcription Kit (Qiagen) following the manufacturer's instructions. Following the RT reactions, cDNA products were diluted five times in distilled water, and 2 µl of the diluted cDNAs was used for PCR using QuantiTect SYBR Green PCR Master Mix and miScript Universal Primer (Qiagen). The PCR reaction was conducted at 95°C for 10 min, followed by 40 cycles at 95°C for 15 s and 60°C for 30 s on a LightCycler 480 real-time PCR machine (Roche). Real-time PCR for mRNAs was performed as described in [47] using the *Rrm2* as a reporter. Differences between samples and controls were calculated based on the 2-ΔΔCP method. Each Real-time PCR reaction was carried out in triplicates using samples from three independent differentiation events of the four ES cell lines (PGK, E14, LF2, HM1). For Figures S1, S2, S3, S4, S5, S6, S7, S8, and S9, Q-RTPCR analyses (miRNA and mRNA) involved two independent differentiation events of pools of female (PGK, XX1 and LF2, XX2) and pools of male (E14, XY1 and HM1, XY2) cell lines, respectively.

### Western Blot of Arid4b protein

Western blotting was performed using standard procedures. The Brcaa1 (Arid4b; ab36962) antibody was purchased from Abcam, (Cambridge, UK).

### Construction of 3′-UTR-luciferase plasmid and reporter gene assays

The 3′ UTR of Arid4b (614 nt) was amplified from DNA extracted from E14 (XY1) ES cells using attB-containing primers (internal primers for the first PCR: Fwd-5′-*AAAAAGCAGGCT* CCATCAATGTCCAGTGCATC-3′, Rev-5′-*AGAAAGCTGGGT*TTTGGTTACCAGGATGATGTCT-3′ and external primers for the second PCR: Fwd-5′-GGGGACAAGTTTGTACAAAAAAGCAGGCT-3′, Rev-5′-GGGGACCACTTTGTACAAGAAAGCTGGGT-3′). The PCR fragment was cloned into the attB-site of pDONR/Zeo (Invitrogen), checked for orientation, sequenced and cloned into the psiCHECK-2 vector (Promega) using Gateway cloning Technology. The resulting plasmid was named psiCHECK-Arid4b.

For reporter assays, HEK-293 cells were transiently transfected with psiCHECK-Arid4b together with miR-302d, miR-291-3p and control siRNA (as indicated in Figure 4D) using lipofectamine 2000 (Invitrogen). Reporter assays were performed 24 h post-transfection using the Dual-luciferase-assay-system (Promega), normalized for transfection efficiency by Renilla-luciferase, also present in psiCHECK-Arid4b. Each experiment was done in triplicate and reproduced twice independently.

### miRNA distribution

The distribution of miRNA frequency was fitted with a Gaussian mixture model. A Bayesian Information Criterion allowed selection of two components. The first component is truncated at zero and corresponds to miRNAs with low counts (interpreted as background). The second component corresponds to miRNAs showing statistically significant expression level. These two components indicated that a reasonable threshold for the “background” expression is T = 0.05%. The dataset was filtered accordingly and all miRNAs with a frequency lower than T were removed from the analysis.

### Correlation of male and female miRNA expression

The similarity of miRNA profiles between the two sexes was tested via a correlation analysis at each time point (0, 2 and 5 days). The Pearson correlation assesses the linear relationship between two variables (here miRNA expression in male XY1 and in female XX1). The Spearman correlation is equivalent to the Pearson correlation, but uses miRNA expression rank as variable. The Pearson correlation therefore assesses similarities in miRNA ranking between male and female samples.

### PAM unsupervised classification

An unsupervised clustering approach was used to group into cluster those miRNAs showing similar expression profiles throughout days 0, 2 and 5 of differentiation. Because miRNA expression data in both sexes are highly correlated, they were used as replicates and averaged for clustering. A partitioning analysis (PAM – partitioning around medoids) was then performed using the Pearson correlation as a measure of similarity. PAM classifies objects in a given number k of groups, each of them being represented by a medoid miRNA indicated in red in Figure 1B. The number of clusters k was chosen as to maximize the average silhouette width of the classes. The silhouette is a measure of the quality of the clustering, based on the difference between the average distance of a given miRNA to all other objects of its class, and the distance between this miRNA to the closest one outside its class.

Calculating the silhouette scores for k = 3 to k = 10, showed that k = 3 achieves the best score. These three classes correspond to a peak of expression at day 0, 2 and 5 respectively. We also tested clustering at a higher resolution (5<k< = 10). In this case, the division into 10 clusters had the best silhouette score and these 10 classes correspond to an exact subdivision of the 3 major classes.

### Significance of PAM classes—Bootstrapping analysis

One thousand randomized datasets were generated from the experimental miRNA expression set (by bootstrapping expression profiles), and the PAM clustering was performed for k = 3. For each dataset, the number of pairs of miRNAs from the same genomic cluster present in the same PAM class was counted. This provided an estimate of the distribution of the number of pairs of neighbour miRNAs in a randomized dataset. The results were then compared to those obtained from the experimental dataset (indicated by a red dot in Figure S7). P-values show that PAM clustering in the experimental data set is very unlikely to occur by chance.

### Differential expression of miRNAs

In order to unravel sex specific miRNAs, we have to take into account that variability of occurrence is smaller for the low-expressed miRNAs than for the high-expressed ones. As a consequence, data were transformed as follows, and represented as MA plot.

M, the ratio of the miRNAs profiles M : log2 (Dxx)−log2 (Dxy)

A, the average miRNAs level, A = (log2(Dxx)+log2(Dxy))/2

The data were split into bins of similar intensities. The number of bins was set to 5 in order to retrieve enough miRNAs so as to estimate the distribution. Outlier miRNAs were identified in each bin by estimating the variance of the bin after discarding the miRNA and then estimating the probability for the miRNA of being an outlier using a Gaussian distribution. All miRNAs with a p-value lower than 5% were judged as significant and considered as being differentially expressed. No multiple testing corrections were applied.

## Supporting Information

Figure S1 Expression of various markers during ES cell differentiation. Time-course quantification of the indicated markers was carried out in 4 distinct ES cell lines in two independent differentiation events. The values obtained for the two independent female cell lines (PGK, XX1; LF2, XX2) and male cell lines (E14, XY1; HM1, XY2) were averaged and are presented here as XX (females) and XY (male). The non-coding Xist RNA is enriched during differentiation only in female cell lines. The slight decrease of the totipotency markers Oct4 and Nanog is consistent with the slow differentiation process induced in mouse ES cells upon LIF removal. Brachyury (mesoderm marker), Fgf5 (Ectoderm marker), and CD34 (Hematopoeïc stem cell markers) have similar profiles during differentiation of each cell line and confirm acquisition of cell identities at D5 of differentiation.
(0.64 MB PSD)

Figure S2 Size distribution and annotation of cloned small RNAs. (A) Size distribution of small RNAs cloned from male (XY1, E14) and female (XX1, PGK) mouse undifferentiated ES cells (D0), after two or five days of differentiation (D2 and D5). The data indicate a population with a median at 22–23 nucleotides. (B) The origin of cloned small RNAs is indicated as a percentage of the total clone number. miRNA: microRNA; rRNA: ribosomal RNA; tRNA: transfer RNA. Other non-coding RNA include scan RNA, as well as small nuclear and small nucleolar RNA, and sequences matching the mitochondrial genome or repeated regions. Other sequences matching the mouse genome are referred to as “genome”, and sequences that cannot be assigned to any of the indicated categories are designated as “not annotated”.
(0.57 MB PSD)

Figure S3 Gaussian mixture model of MicroRNA distribution. The distribution of miRNA frequency was fitted with a Gaussian mixture model. A Bayesian information criterion (BIC) allowed selection of two components. The first component is truncated at zero and corresponds to miRNAs with low counts (interpreted as background). The second component corresponds to miRNAs showing statistically significant expression level. These two components indicated that a reasonable threshold for the “background” expression is T = 0.05% (indicated in red). The dataset was filtered accordingly and all miRNAs with a frequency lower than T were removed from the analysis.
(0.38 MB PSD)

Figure S4 VAMP distribution of cloned miRNAs. The red peaks on each of the 21 chromosomes represent the respective abundance (as expressed as a percentage of total miRNA cloned) for different miRNA clusters. The major expression clusters are indicated by their names on the top of the expression peaks.
(0.84 MB PSD)

Figure S5 Pearson and Sperman correlations between male and female miRNA levels. The similarity of miRNA profiles between male ES cells (XY1: E14 cell line), and female ES cells (XX1: PGK cell line) was tested via a correlation analysis at each time point (0, 2, and 5 days). The Pearson correlation assesses the linear relationship between two variables (here miRNA expression in male and in female). The Spearman correlation is equivalent to the Pearson correlation, but uses miRNA expression rank as variable. The Pearson correlation therefore assesses similarities in miRNA ranking between male and female samples. Note that outlier miRNAs can be seen at D5, mostly corresponding to sex-specific miRNAs.
(0.30 MB PSD)

Figure S6 Partitioning analysis (PAM-partitioning around medoids) of miRNA expression data for k = 10. Clustering analysis for 3<k< = 10 shows that the division into 10 clusters has the best silhouette score. This allows the miRNAs to be grouped into 10 clusters (1 to 10), each characterized by a specific expression profile, which correspond to large subdivisions of the three main PAM classes A, B, and C defined for k = 3, as shown by the hierarchical clustering (top left).
(2.17 MB PSD)

Figure S7 Significance of PAM classes as assessed by bootstrapping. The histogram represents the number of pairs from the same miRNA cluster in the same PAM classes (k = 3) for 1,000 random permutations of all miRNA expression data (bootstrapping). The red point represents the number of miRNAs in the same PAM class as found in the real dataset. The four examples presented here correspond to miRNA clusters containing more than n = 5miRNA, thus allowing pairwise comparisons between cluster members. Cluster names are provided alongside the obtained pvalues, which show that PAM clustering in the real dataset is unlikely to occur by chance.
(0.16 MB PSD)

Figure S8 Expression of selected microRNAs from PAM classes A, B, C. Quantification by QPCR of the indicated microRNA was carried out in 4 distinct ES cell lines in two independent differentiation events. Results for the two independent female cell lines (PGK, XX1; LF2, XX2) and male cell lines (E14, XY1; HM1, XY2) were pooled and are presented here as XX for female cell lines and XY for male cell lines. The miRNAs of each class were chosen so as to not belong to the same genomic clusters.
(4.47 MB PSD)

Figure S9 Putative interactive model between miR-302 and miR-290 families and cell cycle regulation. Recent analyses in human ES cells indicate that the miR-302 promoter is directly activated by the Oct3/4, Sox2, and Nanog transcription factors, which are all required for pluripotency during early embryogenesis and for maintenance of embryonic stem cell identity [1]. Moreover, OCT4 plays also a critical role in pluripotency and cell survival of primordial germ cells [2]. Binding sites for those transcription factors are highly conserved in the core promoter of mammalian miR-302 homologs, including in mouse [3]. The same study identified human Cyclin D1 as a direct target of human miR-302a and showed that expression of miR-302a in primary and transformed cell lines promotes an increase in S-phase and a decrease in G1-phase cells, reminiscent of an ES cell-like cell cycle profile [1]. Accordingly, ectopic expression of miR-302 is sufficient to reprogram human skin cancer cells into a puripotent ES-cell-like state [4]. In the mouse, miR-290 targets key cell cycle regulators (including Cdkn1 or P21) and transcriptional repressors including Rb1, Rbl1, and Rbl2, also resulting in enhanced G1-S phase transition [4]. The data are assembled into a putative model. 1. Card DA, Hebbar PB, Li L, Trotter KW, Komatsu Y, et al. (2008) Oct4/Sox2-regulated miR-302 targets cyclin D1 in human embryonic stem cells. Mol Cell Biol 28: 6426–6438. 2. Anderson R, Copeland TK, Scholer H, Heasman J, Wylie C (2000) The onset of germ cell migration in the mouse embryo. Mech Dev 91: 61–68. 3. Barroso-delJesus A, Romero-Lopez C, Lucena-Aguilar G, Melen GJ, Sanchez L, et al. (2008) Embryonic stem cell-specific miR302-367 cluster: human gene structure and functional characterization of its core promoter. Mol Cell Biol 28: 6609–6619. 4. Lin SL, Chang DC, Chang-Lin S, Lin CH, Wu DT, et al. (2008) Mir-302 reprograms human skin cancer cells into a pluripotent ES-cell-like state. RNA 14: 2115–2124.
(0.25 MB PSD)

Dataset S1 Set of cloned microRNAs. Sheet 1 contains percentage of cloned microRNAs per library. Sheet 2 contains sequences of 17 new miR* star (red) and their position in the precursor sequence.
(0.15 MB XLS)

Dataset S2 Predicted target transcripts from the EIMMo target prediction software for miR-290 cluster (Sheet 1), miR-293 (Sheet 2), and miR-302 cluster (Sheet 3).
(0.20 MB XLS)

Dataset S3 Predicted target transcripts from the Pictar target prediction software for miR-290 cluster (Sheet 1), miR-293 (Sheet 2), and miR-302 cluster (Sheet 3).
(0.18 MB XLS)
