# Correction: Highly Dynamic and Sex-Specific Expression of microRNAs During Early ES Cell Differentiation

**DOI:** 10.1371/journal.pgen.1005077

**Published:** 2015-03-25

**Authors:** The PLOS Genetics Staff

Fig. 3 is incorrect. A mistake was made by the authors during the assembly of panel 3B. The loading control U6 is duplicated for the XY D0 and D2 samples. The authors apologize for this error and have provided a corrected version here.

**Fig 3:**
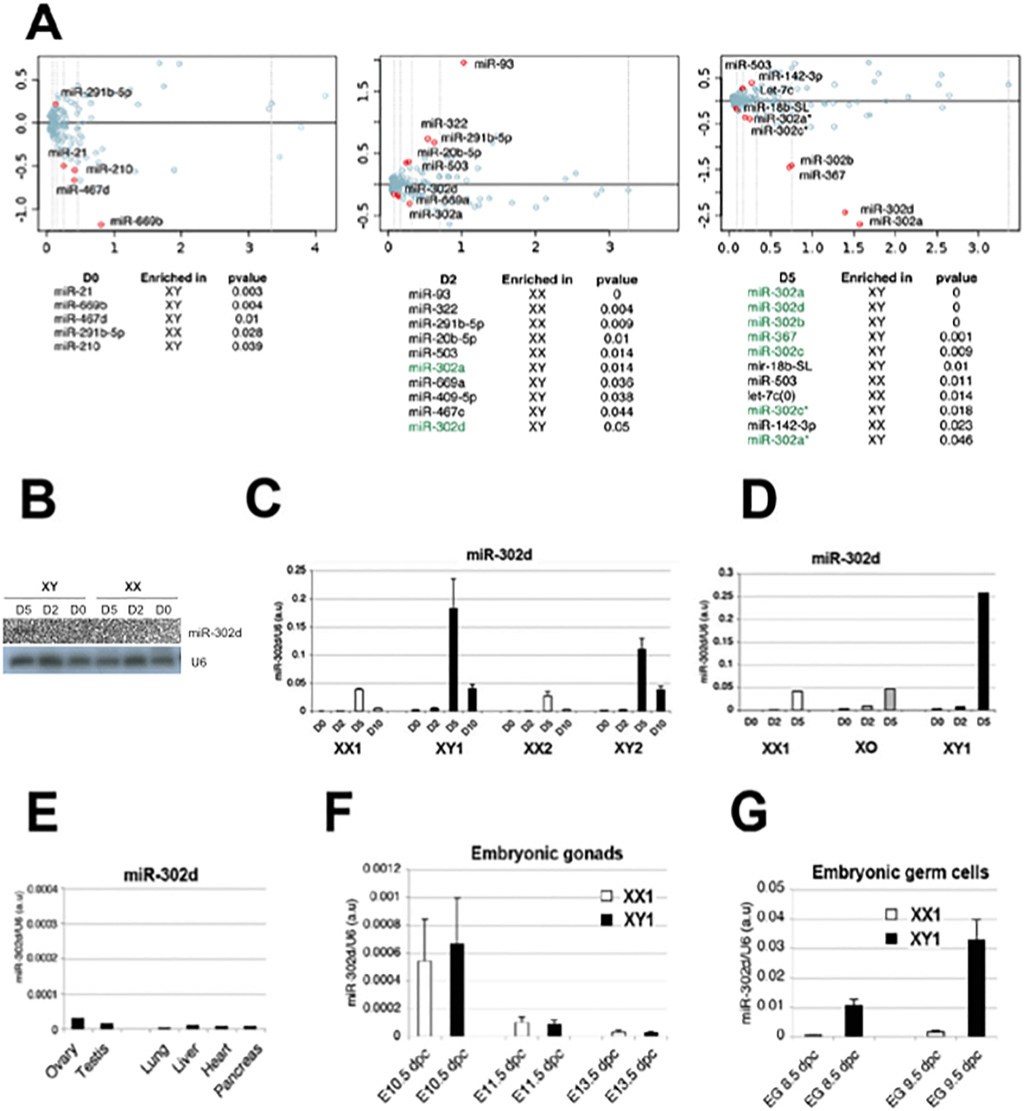
Male-specific regulation of the miR-302 gene in embryonic stem cells and germ cells. (A) MA Plot of microRNAs singularizes those enriched in male (E14, XY1) or female (PGK, XX1) cells at different time points, as indicated in red (see Materials and Methods for details). (B) Northern Blot analysis of miR-302d in female (XX1; PGK) and male (XY1; E14) cell lines at D0, D2, and D5 of differentiation. (C,D) Quantification by QRT-PCR analysis of mature miR-302d in two independent female (PGK, XX1 and LF2, XX2) and male (E14, XY1 and HM1, XY2) ES cell lines at D0, D2, D5, and D10 of differentiation (C) and in the XPGKO ES cell line (D). (E) Quantification by RT-PCR of miR-302d accumulation in various adult mouse tissues. (F,G) Quantification by RT-PCR of mir-302d expression in male (XY, black) and female (XX, white) gonads at 10.5 to 13.5 dpc (F) and 8.5 to 9.5 dpc. (G) Same as in (F), but in male (XY, black) and female (XX, white) embryonic germ cells (EG).
